# Indian Expert Consensus on the Selection of Vascular Access Devices in Oncology Patients

**DOI:** 10.1007/s13193-025-02466-7

**Published:** 2025-11-25

**Authors:** Somashekhar S. P., Bharat Gupta, D. G. Vijay, Geeta Kadayaprath, Manjiri Somashekhar, Manjula Rao, Rahul Chowdhary, Raghunandan Prasad, Sanjeev Kumar Patni, Shirish S. Alurkar, Mohammad Jahanzeb

**Affiliations:** 1 Aster International Institute of Oncology, Bangalore, India; 2 Rajiv Gandhi Cancer Hospital, New Delhi, India; 3 HCG Aastha Cancer Centre, Ahmedabad, India; 4 Apollo Hospitals, Delhi, India; 5 Aster Hospitals, Bangalore, India; 6 Apollo Proton Cancer Centre, Chennai, India; 7 Wockhardt Hospital, Rajkot, India; 8 Amrita School of Medicine, Amrita Vishwa Vidyapeetham, Faridabad, India; 9 Bhagwan Mahaveer Cancer Hospital and Research Centre, Jaipur, India; 10 OncAdvisor, Boca Raton, USA

**Keywords:** Central venous access devices, Infusion ports, Cancer chemotherapy, Central venous catheterization, Practice guidelines, Chemoport

## Abstract

To develop consensus recommendations for the selection and management of vascular access devices (VADs) in oncology patients in India by addressing unique challenges in the Indian healthcare ecosystem. An expert panel of 11 specialists in oncology and interventional radiology convened to review literature and develop consensus statements on 10 key questions related to the selection of VAD in cancer patients. The panel used a nominal group technique during a face-to-face meeting, achieving 100% consensus on all statements through structured discussions. The panel deliberated and agreed upon 10 consensus statements, addressing the indications for central venous access devices (CVADs), available CVAD options, clinical decision-making processes for identifying appropriate CVADs for different groups of patient, decision-makers for device selection and insertion, factors influencing port selection, the logistical requirements and CVAD maintenance protocols in cancer patients in India. Key recommendations emphasize the use of CVADs for administering vesicant drugs, considering both external catheters and implantable ports based on the duration and frequency of treatment. The recommendations also highlight the importance of involving multidisciplinary teams in device selection and implementing standardized training and protocols for insertion and maintenance. These consensus recommendations provide guidance on VAD selection and management tailored to the Indian oncology setting. The statements emphasize the importance of patient-centred decision-making, proper training for healthcare providers, and the need for increased awareness and education to enhance the appropriate adoption of implantable ports in India. Implementation of these recommendations may improve vascular access care and outcomes for cancer patients across diverse healthcare environments in India.

## Introduction

The global burden of cancer continues to rise, with 19.3 million new cases and 10.0 million deaths in 2020, projecting a 57.5% increase by 2040.[1–3] This growing patient population presents significant challenges for oncology care, as up to 75% of these patients require intravenous therapies for chemotherapy, supportive care, transfusions, administration of antimicrobial agents, parenteral nutrition, and diagnostic sampling.[1] Chemotherapy administration requires an intravenous access that is safe, reliable, re-accessible, comfortable and cost-effective for the patient, as well as devices which are easy to insert, maintain, and remove for the healthcare providers.

Vascular access devices (VADs) are a cornerstone of cancer management in all settings, including curative, when it involves prolonged intravenous systemic therapy, and increasingly prolonged palliative settings.[4] Selecting the appropriate VADs is important, as repeated venipuncture, chemotherapy-induced vascular damage, and thrombotic events can progressively deplete usable veins.[4, 5] For cancer patients receiving chemotherapy, central venous access is preferred over peripheral routes, because many antineoplastic drugs are vesicants or irritants that can cause serious complications such as extravasation, tissue damage, and damage of peripheral veins. Although peripheral infusion is still common in some settings, current standards recommend central access, such as a peripherally inserted central catheter (PICC) or a totally implantable port for vesicant drug administration. [4, 6, 7].

In India, the available central venous access devices (CVADs) for cancer patients include PICCs, tunnelled catheters, and totally implantable ports. Planning for vascular access should include selection of the most appropriate VAD, minimizing vascular harm, and preserving veins for future medical needs.[8] Despite the availability of various device types, optimal device selection remains challenging due to a lack of evidence-based guidance suited to the Indian healthcare setup. Most data guiding VAD choice are derived from qualitative studies, which makes it difficult for clinicians to provide consistent standards of vascular access care.

Existing international guidelines from the American Society of Clinical Oncology (ASCO)[9], European Society for Medical Oncology (ESMO)[6], and Infusion Nurses Society (INS)[7] provide recommendations for device selection, placement, and complication management. A recent international expert panel consensus has provided pragmatic recommendations on indications, maintenance, and logistics of CVAD use in oncology.[10] However, its direct applicability to the Indian healthcare ecosystem may be limited by unique challenges such as resource constraints, variability in healthcare provider expertise, and cost considerations.[11–13] For example, guidance on device maintenance, flushing schedules, and infection prevention requires the availability of disposables, specialist staff, and robust laboratory support, which is not always available across Indian oncology units. Complications such as catheter-related bloodstream infections (CRBSI), mechanical failures, infiltration, occlusion, and thrombosis are also observed in clinical practice, causing therapy interruptions, increased healthcare costs, and poor patient outcomes.[11, 12].

These circumstances provided the rationale for this Indian expert panel to convene and critically evaluate how best to select and manage VADs within the country’s diverse clinical settings. The panel considered patient profiles, disease characteristics, treatment regimens, care and maintenance protocols, logistical challenges, and economic factors, with the goal of developing consensus recommendations that are relevant and implementable across India's diverse healthcare environments. This article presents the consensus reached by the Indian expert panel, providing guidance on VAD selection, device maintenance, logistical management, and cost-effective practices for the Indian oncology community.

## Methods

### Panel Composition

This consensus initiative included 11 experts with extensive experience in CVAD management of cancer patients. The panel comprised of five surgical oncologists, three medical oncologists, two interventional radiologists and one paediatric surgical oncologist. All participating clinicians had a minimum of ten years of specialized experience in CVAD selection, insertion, and maintenance in oncology practice settings.

### Literature Review

A systematic literature search was conducted across four major databases, PubMed, Embase, Cochrane Library, and Google Scholar, to identify relevant evidence regarding CVAD utilization in adult cancer populations. The search encompassed publications published from 1990 to May 2025. The search strategy incorporated multiple term categories. Device-specific terminology included "peripherally inserted central catheter," "PICC," "totally implantable venous access port," "port-a-cath," “chemoport”, "tunnelled central venous catheter," "non-tunnelled central venous catheter," "Hickman catheter," "Groshong catheter," and "central venous access device." Cancer-related terms included "oncology," "malignancy," "neoplasm," "chemotherapy," "systemic therapy," "hematologic malignancy," "solid tumor," and "cancer patient.". Clinical outcome measures were recorded using terms such as "catheter-related bloodstream infection," "venous thromboembolism," "catheter occlusion," "mechanical complications," "device failure," "patency," and "catheter dysfunction.". Procedural aspects were addressed through "catheter insertion," "vascular access placement," "catheter removal," "ultrasound guidance," and "fluoroscopic guidance.". Maintenance protocols were identified using "catheter care," "flushing protocols," "heparin lock," "saline lock," "catheter maintenance," and "infection prevention." Additional search terms included "quality of life," "patient satisfaction," "cost-effectiveness," "healthcare utilization," "multidisciplinary team," "clinical guidelines," and "best practices.". Boolean operators (AND, OR) were employed to combine terms effectively across PubMed, Embase, and Cochrane Library, while Google Scholar searches utilized natural language queries. Citation tracking was performed manually to identify additional grey literature and institutional guidelines. Reference lists of the included studies and relevant systematic reviews were manually searched to identify additional pertinent literature. All searches were limited to human studies in English. Two independent reviewers performed data extraction and article relevance assessments. The synthesized evidence was compiled into a comprehensive presentation for discussion during the expert panel meet.

### Question Development and Selection

The consensus addressed ten questions adapted from a recently published international expert consensus on CVAD practice(10), selected because of their broad applicability to adult cancer patients across diverse healthcare settings in India. Each question addressed critical aspects of clinical decision-making, including indications for CVAD placement, criteria for selecting the appropriate device for relevant patient groups, responsibility for insertion procedures, logistical prerequisites, maintenance protocols, and strategies for the enhanced adoption of port systems within the Indian healthcare context.

### Consensus Development Process

The consensus methodology employed a nominal group technique conducted during a face-to-face meeting held on July 5, 2025, in Bengaluru, India. During the session, the individual panellists shared their institutional practices and clinical experiences relevant to each question. This was followed by the presentation of evidence synthesis that was subject to discussion among the experts. For each question, the steering committee, comprising two designated moderators, both of whom had previously participated in the Global Consensus Panel’s recommendations that are already published(10) presented initial draft statements that were subsequently reviewed and refined through systematic round-robin discussion. This iterative process ensured statement clarity, clinical accuracy, and comprehensive coverage of relevant considerations. Through structured dialogue and collaborative refinement and rephrasing, all 11 panellists achieved unanimous agreement (100% consensus) on each finalized statement.

### Documentation and Validation

All consensus statements and accompanying expert insights were documented in real-time throughout the meeting proceedings. Upon completion of the deliberations for each question, the finalized statements were presented to the entire panel for formal ratification. The complete transcripts and all presentation materials were archived to ensure process transparency and reproducibility. The achievement of a full consensus during the initial nominal group session eliminated the need for subsequent Delphi rounds or additional validation procedures.

## Results and Discussion

**Fig. 1:**
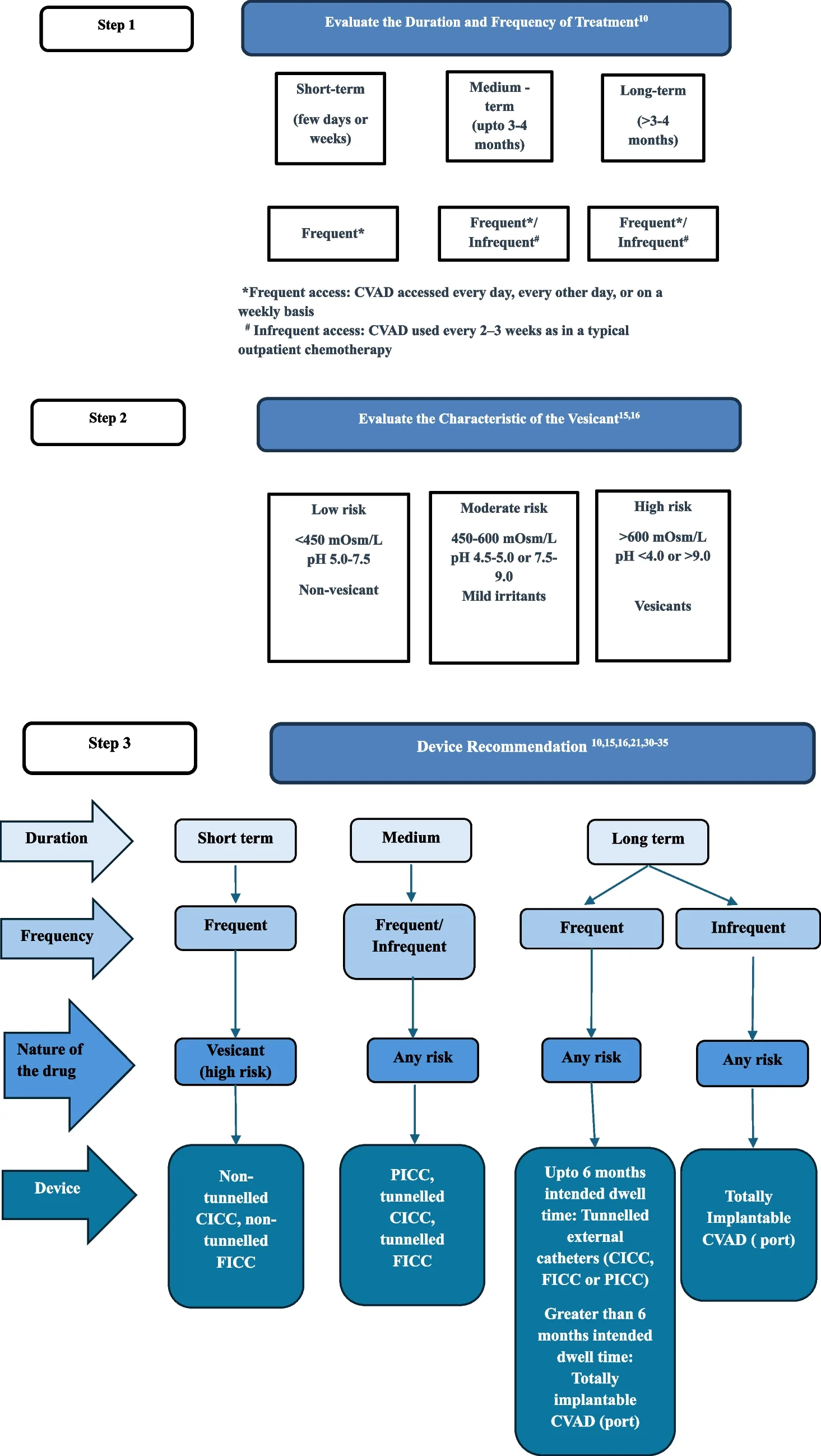
Algorithm for CVAD selection in adult cancer patients. Abbreviations: CICC (centrally inserted central catheter), CVAD (central venous access device), FICC (femorally inserted central catheter), ICU (intensive care unit), PICC (peripherally inserted central catheter)

The panel deliberated upon carefully chosen ten consensus statements and all of them received 100% agreement from the expert panel, reflecting a strong clinical consensus across diverse oncology subspecialties and institutional settings. The final recommendations are presented in Table 1. It was noted that the consensus closely aligned with the global consensus(10), with some nuances specific to the Indian circumstances. During the consensus meeting, the expert panel collaboratively developed a clinical decision-making algorithm to guide appropriate CVAD selection for cancer patients in an Indian healthcare setting. This algorithmic framework (Fig. 1) includes the key determinants identified in the consensus recommendations. It offers a systematic method for selecting devices based on the expected duration and frequency of vascular access needs, as well as the type and vesicant properties of the planned chemotherapy regimens.

**Table 1:** Summary of Consensus recommendations

**1**	**What are the indications for a CVAD in Indian cancer patients?** CVADs are strongly recommended when feasible/possible for infusing chemically irritating or vesicant drugs to reduce the risk of peripheral phlebitis and/or extravasation. Also, CVADs should be considered when there is insufficient peripheral venous access for the duration of the planned treatment or in the context of failed or difficult peripheral intravenous access, despite peripherally compatible infusate.
2	**What are the CVAD options for cancer patients in India?** Both external catheters (tunnelled and non-tunnelled CVADs like PICC, CICC, or FICC based on the insertion site) and totally implantable devices (ports including chest, brachial, and femoral ports) should be considered for patients with cancer, with the choice depending on patient needs, treatment duration, and clinical setting.
3	**Who makes the decision regarding the choice of CVAD?** The selection of the most appropriate CVAD should be a collaborative process, involving the primary treating physician, vascular access specialists, patient, device maintenance team, and, when applicable, the patient’s caregiver. The choice must be based on a comprehensive understanding of the patient’s condition, the device and its insertion techniques, potential complications, and suitability of the device to the intended duration of therapy and medications.
4	**Who is responsible for inserting the CVAD?** **Ports:** Healthcare professionals with specific and appropriate training within the regulatory framework of the relevant healthcare system should insert the port. This includes physicians (surgeons, interventional radiologist, anaesthetists, oncologist, intensivist, and any other duly trained physician). **External catheters (PICC, FICC, CICC):** Healthcare professionals with suitable and specific training within the regulatory framework of the relevant healthcare system, including physicians (such as surgeons, interventional radiologists, anaesthetists, oncologists, intensivists, and other duly trained physicians), advanced practice clinicians (like nurse practitioners and physician assistants), and staff nurses can insert external catheters if permitted by institutional protocols after due training.
5	**Which factors determine the choice of a port?** Totally implantable ports are preferred for long-term vascular access (> 3–4 months duration, access every 2–3 weeks). For patients with cosmetic concerns or those engaging in water activities, ports are preferred as they offer psychological advantages and less lifestyle disruption. In settings where regular access to central lines is challenging due to environmental, logistical, or personal factors ports may provide additional advantage, offering easier maintenance and a lower infection risk.
6	**What are the prerequisites for the insertion of the port in terms of logistics in India?** The prerequisites for the insertion of a port in terms of logistics include (a) availability of the device and access to proper facilities; institutions should be strongly encouraged to have a policy for VAD,including essential equipment and supplies for asepsis, imaging, and monitoring devices, and effective management of biomedical waste and (b) availability of clinicians or specialized vascular access teams with specific training in venous access procedures. The best practice is to follow image guided placement (ultrasound guidance for access and fluoroscopic/X-ray guidance to confirm the tip position).
7	**What are the prerequisites for the insertion of other CVADs in terms of logistics in India?** The logistical prerequisites for inserting an external catheter include device availability, provider and staff competence with specific training in VAD management, access to adequate facilities and appropriate equipment, and informed patient consent.
8	**Which factors determine the removal of a port after the completion of definitive therapy?** Ports should be removed after completion of definitive therapy in all patients with a low risk of early relapses and favourable prognosis. However, patients with high risk of early relapses and unfavourable prognosis should retain the port with regular recommended flushing protocol.
9	**How frequently should a central line be flushed if not in use?** If not in regular use, ports should be flushed with normal saline every 3 months or according to the instruction for use; external catheters require weekly flushing. Anticoagulants are not recommended unless indicated for other clinical conditions.
10	**How do we enhance port adoption in India?** The panel unanimously agrees that implantable ports are more cost-effective in the long term, despite the initial cost, and result in better overall outcomes for the patient compared to other CVADs. There is a need to increase awareness among healthcare providers and patients with better training and education, as well as the creation of appropriate referral pathways.

^Abbreviations^^: C^^ICC (centrally inserted central catheter), CVAD (central venous access device), FICC (femorally inserted central catheter), PICC (peripherally inserted central catheter), VAD (venous access device)^

## 1. What are the Indications for a CVAD in Indian Cancer Patients?

CVAD selection in patients with cancer requires careful evaluation of multiple clinical factors. The primary determinant involves anticipated therapy duration, with devices classified as short-term (days to weeks), medium-term (up to 3–4 months), or long-term (exceeding 3–4 months), and the access frequency patterns.[4, 10] The administration of irritant or vesicant agents through peripheral access carries substantial risks including extravasation, and progressive venous depletion, with reported incidence rates varying from 0.01% to 7% across studies.[6] While the occurrence of extravasation through CVAD is relatively rare compared to peripheral routes, the potential for tissue damage can vary greatly depending on whether the drug is classified as a vesicant, irritant, or a non-vesicant.[6, 10, 14–16] Knowing the pH and osmolarity of intravenous drugs is important as studies show that endothelial damage begins to occur at 600 mOsm/L. [7, 15, 17] Hence, medications with osmolarity > 600 mOsm/L are classified as vesicants and CVADs are recommended for both boluses and continuous infusions of these vesicant chemotherapeutic drugs.[7, 15, 16, 18] When considering pH levels, experimental data suggest that peripheral veins can tolerate solutions with a pH between 5.0 and 7.5. However, for vesicant drugs with extreme pH values below 4.0 or above 9.0, central access is required due to the significant risk of endothelial injury.[15, 16].

In addition to the duration of therapy and the type of infusate, central venous access is also indicated when peripheral venous cannulation is compromised by factors such as advanced age, scarring, obesity, peripheral vascular disease, or other conditions that limit the accessibility of superficial veins.[7, 10] While ultrasound-guided peripheral VADs are suitable for non-irritant infusions, central access is preferred when repeated peripheral attempts fail or when the treatment duration exceeds peripheral device capabilities.[7] Quality of life considerations make central venous access particularly valuable for patients receiving ambulatory chemotherapy or palliative care. These devices enhance patient comfort by enabling outpatient and home-based therapies, while providing clinicians with reliable central access.[10].

### Indian Expert Panel Consensus

CVADs are strongly recommended when feasible/possible to infuse chemically irritating or vesicant drugs to reduce the risk of peripheral phlebitis and/or extravasation. In addition, CVADs should be considered when there is insufficient peripheral venous access for the duration of the planned treatment or in the context of failed or difficult peripheral intravenous access, despite peripherally compatible infusate.

### Experts’ Opinion:

Central venous access is essential in the management of cancer patients, as it minimizes vascular trauma from repeated peripheral cannulations, reduces the risk of access failure, improves patient comfort, prevents extravasation injuries due to vesicant drugs and preserves venous integrity for future treatment lines.

#### Disease-Specific Recommendations for CVAD:

- Solid tumours: Ports are preferred for long-term therapy and lifestyle compatibility.
- Breast cancer: CVADs are indicated for TNBC, HER2 + , bilateral disease, recurrent/metastatic cases where peripheral veins are often compromised from previous treatments, necessitating central venous access. Ports are preferred in these patients.
- Colorectal cancer, locally advanced and metastatic disease: Ports are preferred in these patients especially if infusional chemotherapy (5-fluorouracil) is contemplated.
- Hematological malignancies: PICC lines are preferred for frequent monitoring and blood sampling; Hickman catheters are reserved for bone marrow transplantation.

#### Vulnerable Patient Populations Requiring CVAD:

- Pediatric patients with solid malignancies requiring extended treatment protocols.
- Elderly patients over 70 years old who are receiving palliative chemotherapy or home-based infusions often face challenges with peripheral access due to age-related vascular changes.
- Gastrointestinal cancer patients requiring prolonged continuous infusions (5-FU-based regimens).
- Patients receiving immunotherapy or intravenous targeted therapies.
- High-risk/advanced malignancies requiring intensive treatment protocols with multiple agents.
- Post-axillary surgery in patients with bilateral breast cancer when both arms are contraindicated for peripheral access due to lymphedema risk.

#### Treatment-Specific Indications for CVAD:

- Vesicant drugs administered for more than 3 cycles.
- Chemotherapy regimens containing vesicant drugs (anthracyclines).
- Treatment durations exceeding 3 months or 6 cycles.

#### Patient-Related Factors for CVAD:

- Active patients need CVADs that provide reliable access with minimal maintenance.
- Patients hailing from rural areas and low-income patients who have logistical barriers to device access and maintenance also need CVADs.

#### Healthcare Infrastructure and Availability:

CVAD is needed when healthcare infrastructure includes an expert team for insertion, maintenance, and management of complications.

#### Pediatric Considerations:

 Ports are commonly preferred in pediatric patients requiring long-term venous access, particularly for extended treatment courses. Their use is associated with higher acceptance among active children and minimal disruption to daily activities. In some pediatric centers, Groshong ports are used in approximately 40% of cases, potentially due to limitations in size availability or clinician preference. PICC lines provide advantages in easier insertion/removal, lower upfront costs, and simplified infection management. Subclavian access using the Seldinger technique is preferred. Jugular access is suitable for patients > 2 years with adequate neck length. Femoral access is avoided due to contamination risks. Infection diagnosis in pediatric cancer patients with CVADs is challenging due to uncertainty whether infections are device-related or from treatment-induced immunosuppression. While cultures can be obtained through ports, PICC lines offer diagnostic advantages as they can be removed to assess the device's role in infection.

## 2. What are the CVAD Options for Cancer Patients in India?

Commonly used CVADs include nontunnelled central venous catheters (CVCs), PICCs, totally implantable ports, and tunnelled CVCs.[19] Nontunnelled CVCs are best suited for short-term inpatient use when peripheral access is contraindicated. Peripheral cannulas or midline catheters should be reserved for non-vesicant therapies, isotonic parenteral nutrition, and solutions with a pH between 5 and 9.(4) Tunnelled CVCs are indicated for patients requiring prolonged central access with repeated chemotherapy infusions, antibiotic therapy, parenteral nutrition, blood product transfusions, or frequent laboratory sampling.[4] PICCs are used in cancer care, allowing bedside insertion by trained nurses.[20] Totally implantable ports provide long term access, with low maintenance burden and discreet appearance.[21, 22] Ports show the lowest catheter-related bloodstream infections per 1,000 catheter-days and fewer thrombotic complications.[19, 23, 24] For anticipated treatment durations of 3–4 months with frequent access requirements, external catheters like PICCs and tunnelled CVCs are preferred. When therapy is expected to extend beyond this timeframe and is administered every two to three weeks, ports are the preferred choice.[10].

### Indian Expert Panel Consensus:

Both external catheters (tunnelled and non-tunnelled CVADs, such as PICC, CICC, or FICC, based on the insertion site) and totally implantable devices (ports, including chest, brachial, and femoral ports) should be considered for patients with cancer, with the choice depending on patient needs, treatment duration, and clinical setting. (Fig. 1).

### Experts’ Opinion:

Central venous access device selection is guided by two key parameters: treatment duration and access frequency. Duration of therapy is categorized as short-term (few days to weeks), medium-term (up to 3–4 months), and long-term (> 3–4 months). Frequency of access is defined as frequent (daily, every other day, or weekly usage) or infrequent (every 2–3 weeks). (Table 2).

**Table 2:** Selection of CVAD based on anticipated duration of therapy

Treatment Duration	Appropriate CVADs
**Short-term** (few days or weeks) **Frequent access** ((used every day, every other day, or on a weekly basis)	Non-tunnelled CICCs Non-tunnelled FICCs
**Medium-term** (up to 3–4 months) **Frequent/infrequent access**	PICCs Tunnelled CICCs Tunnelled FICCs
**Long-term** (> 3–4 months) **Frequent access**	Up to 6 months intended dwell time^21^: Tunnelled external catheters (PICCs, CICCs, or FICCs) Greater than 6 months intended dwell time: Totally implantable CVAD (ports)
**Long-term** (> 3–4 months) **Infrequent access** (every 2–3 weeks (as in a typical outpatient chemotherapy)	Totally implantable CVAD (ports)

^Abbreviations^^:^^*CICC* centrally inserted central catheter, *CVAD* central venous access device, *FICC* femorally inserted central catheter, *PICC* peripherally inserted central catheter^

**‘**

## 3. Who Makes the Decision Regarding the Choice of CVAD?

**Fig. 2:**
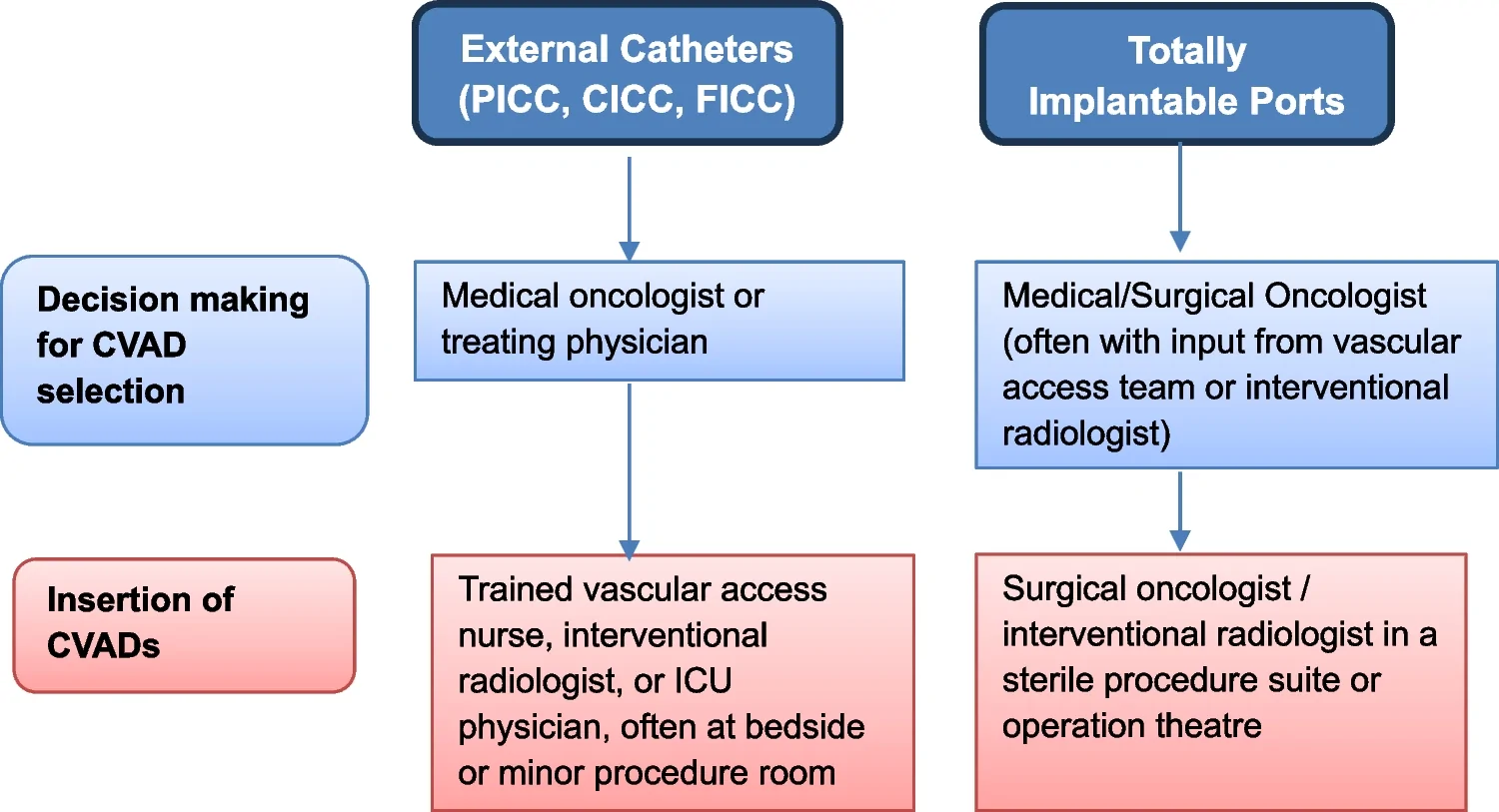
Stakeholders involved in CVAD selection and insertion. Abbreviations: CICC (centrally inserted central catheter), CVAD (central venous access device), FICC (femorally inserted central catheter), ICU (intensive care unit), PICC (peripherally inserted central catheter)

The referring healthcare provider is usually the primary decision-maker, guided by the patient's clinical needs and therapy requirements, such as chemotherapy, parenteral nutrition, or long-term medication.[25, 26] Decision quality is enhanced with multidisciplinary collaboration, as vascular access nursing specialists and dedicated teams can provide essential assessments of venous patency and insertion techniques.[7, 10] Therefore, the choice of CVAD should involve multiple key stakeholders: the referring clinician, the healthcare provider performing the procedure, the patient, and device maintenance staff. (Fig. 2) A comprehensive decision process should also consider the device availability, individual patient preferences and the patient’s or caregiver’s ability to maintain the device.[4] Institutional protocols and decision-support tools help standardize the selection process, clearly define selection criteria and establish an escalation pathway that requires senior-level consultation for high-risk cases. This approach has been shown to reduce insertion failures and lower infection rates.[26].

### Indian Expert Panel Consensus:

The selection of the most appropriate CVAD should be a collaborative process involving the primary treating physician, vascular access specialists, patient, device maintenance team, and, when applicable, the patient’s caregiver. The choice must be based on a comprehensive understanding of the patient’s condition, the device and its insertion techniques, potential complications, and the suitability of the device for the intended therapy and medications.

### Experts' Opinion:

In the clinical workflow, the healthcare provider who first encounters the patient and evaluates their vascular access needs often guides the decision-making process. This provider could be a medical or surgical oncologist. Ideally, this initial decision should involve consultation with the broader care team, including interventional radiologists, vascular access specialists, nursing staff, and other relevant experts who can offer valuable insights into device selection based on their expertise. Another ideal scenario would be to discuss CVAD selection during tumor board meetings. However, the reality of clinical practice often involves time constraints, urgent treatment timelines, and immediate clinical needs that require rapid decision-making. Consequently, the initial healthcare provider (medical/surgical oncologist) makes the choice the CVAD selection. A critical gap in current practice is the systematic exclusion of patients and their caregivers from this decision-making process, despite the significant impact CVAD selection has on quality of life, daily activities, body image, and long-term care management. It is observed in clinical practice that patients who understand their options, participate in decision-making, and have realistic expectations about device management are more likely to demonstrate better compliance, report higher satisfaction, and experience an improved quality of life throughout their treatment journey.

## 4. Who is Responsible for Inserting CVADs?

Clinicians responsible for inserting totally implantable ports and PICCs must undergo comprehensive, device-specific training and demonstrate procedural competence in line with their healthcare system's regulatory standards. This group includes physicians (such as surgeons, interventional radiologists, anaesthetists, oncologists, intensivists, and other appropriately trained doctors), advanced practice clinicians (nurse practitioners, physician assistants), and staff nurses.[10] (Fig. 2) Guidelines stipulate that operators must receive formal didactic instruction, perform supervised insertions, and pass objective skills assessments before independently performing these procedures. Additionally, annual competency validation and ongoing continuing education are required to maintain best practices.[7, 14, 27].

Typically, PICC placement is conducted at the patient's bedside by trained physicians or nurses, using either "blind" insertion techniques through the antecubital or cephalic veins or ultrasound-guided methods via deeper mid-arm vessels like the basilic or brachial veins.[4] Healthcare providers using two-dimensional ultrasound for central venous catheter placement must be adequately trained to ensure the safe and effective use of this technology.[27] Structured ultrasound education programs significantly reduce both infectious and mechanical complications associated with inexperienced operators and decrease repeated venipuncture attempts.[28] Educational courses incorporating simulator models for psychomotor feedback are recommended to develop essential ultrasound-guided cannulation skills.[29].

### Indian Expert Panel Consensus:

Ports: Healthcare professionals with specific and appropriate training within the regulatory framework of the relevant healthcare system should insert a port. This includes physicians (surgeons, interventional radiologists, anaesthetists, oncologists, intensivists, and any other duly trained physician).

External catheters (PICC, CICC, FICC): Healthcare professionals with suitable and specific training within the regulatory framework of the relevant healthcare system, including physicians (such as surgeons, interventional radiologists, anaesthetists, oncologists, intensivists, and other duly trained physicians), advanced practice clinicians (such as nurse practitioners and physician assistants), and staff nurses, can insert external catheters if permitted by institutional protocols after due training.

### Experts’ Opinion:

In India, port insertion practices differ between surgical specialties. Breast surgeons often place ports during the initial surgery as conditions are sterile, patient is under anaesthesia, and most patients have good prognosis, with many requiring post-operative intravenous adjuvant therapy. However, such systemic therapy is now increasingly being initiated pre-operatively. Combining tumour removal and port placement in a single operation simplifies patient care. However, surgeons handling abdominal or gastrointestinal tumours avoid port insertion during primary surgery due to non-sterile conditions from bowel exposure, which increases infection risk. Trained personnel must perform port insertions. Surgical oncologists and interventional radiologists predominantly insert ports, with medical oncologists handling select cases. The panel acknowledges that while various trained healthcare provides can perform PICC insertion, the nursing-led approach dominates in Indian healthcare. The panel noted that trained nurses with institutional credentialing demonstrate excellent competency in PICC insertion, achieving comparable success rates. However, this requires proper protocols, training programs, and supervision to ensure safety and quality (Fig. 2).

## 5. Which Factors Determine the Choice of a Port?

The selection of a port for venous access is based on patient factors, clinical needs, available resources, and staff expertise. Port selection over external catheters is guided by anticipated duration and frequency of vascular access needs. Ports are preferred for long-term venous access exceeding 3–4 months, with access every 4 weeks, matching outpatient chemotherapy schedules for solid tumours.[4, 10, 30–35] Unlike external catheters requiring weekly maintenance, ports need flushing every 4–12 weeks, making them suitable for extended treatment intervals.[10].

Ports are commonly implanted early in chemotherapy to avoid venous access difficulties later in treatment.[4] Ports are recommended for administering vesicant or irritant chemotherapeutic agents, to reduce the risk of local tissue injury and complications associated with peripheral administration.[7, 10, 19, 36, 37] Ports improve the quality of life of cancer patients,[19, 38, 39] and have fewer overall adverse events, including lower rates of catheter-related thrombosis (OR = 2.84, 95% CI = 1.97–4.11)[40] when proper insertion techniques are used, including ultrasound guidance and appropriate catheter-to-vein ratios.[41] Ports reduce contamination risks during routine activities, making them convenient for nonhospitalized patients receiving outpatient therapy. [10] Ports offer a superior safety profile, particularly regarding infection, over external catheters for long-term use.[31, 41, 42] Despite these advantages, certain patient factors, such as coagulation disorders or bleeding risks, present relative contraindications to port placement.[10, 43] Port selection requires careful consideration of institutional resources, access to proper surgical facilities with imaging capabilities, and trained healthcare professionals skilled in port placement and maintenance. These aspects are discussed in the subsequent sections.

Cost is a major consideration in port use.[11] VAD-related complications can incur treatment costs up to US$56,000 and extend hospital stays by 20 days.[44–47] Frequent PIVC replacements add significant expenses, each costing up to US$35 in the US and €10.84 in Europe.[44, 48, 49] Ports reduce these costs in long-term by lowering complication rates and the need for device replacements. Outpatient port procedures further save costs and time compared with inpatient care. A randomized trial of 399 cancer patients showed higher healthcare costs for PICCs than ports, mainly due to adverse event management.[50] A cost-consequence analysis showed that ports had a lower overall complication rate than both Hickman (incidence rate ratio 0.422; 95% CI 0.286–0.622) and PICC (incidence rate ratio 0.295; 95% CI 0.189–0.458), and were less likely to require unplanned device removal. The total cost of the device in situ was lower for ports than for Hickman (− £98.86; 95% CI − 189.20 to − 8.53) and similar to that of PICC (− £48.57; 95% CI − 164.99 67.86). No differences were observed in chemotherapy interruptions or health utilities. Although the insertion of ports was more expensive, when time on device was considered, the mean total cost of a port was lower than that of a Hickman and comparable with PICC, making ports more effective and less costly than Hickman and cost-effective compared to PICC for long-term (≥ 12 weeks) anticancer therapy in solid malignancy. [51] The use of ports also reduces direct medical care costs compared to PICCs, yielding cost savings for hospitalized cancer patients needing CVADs.[52].

### Indian Expert Panel Consensus:

Totally implantable ports are preferred for long-term vascular access (> 3–4 months, access every 2–3 weeks). For patients with cosmetic concerns or those engaging in water activities, ports are preferred because they offer psychological advantages and reduce lifestyle disruption. In settings where regular access to central lines is challenging owing to environmental, logistical, or personal factors, ports may provide additional advantages, offering easier maintenance and a lower infection risk.

### Experts’ Opinion:

The panel recommends inserting ports before first-line chemotherapy to optimize treatment delivery, minimize delays, reduce complications from emergency port placement during active treatment and improve cost-effectiveness. This approach allows proper healing and device stability before the initiation of intensive therapy. Proper technique is crucial for achieving optimal outcomes. The panel recommends the use of monofilament sutures rather than absorbable sutures to prevent port rotation over time. Suture lines should be positioned away from the port reservoir to minimize wound healing complications and infection risks. Operator experience and adherence to appropriate procedural protocols are critical for success. The panel also addressed several procedure-specific challenges that occur during port insertion, including achieving the optimal length and malleability of tunnelling devices, using atraumatic needles to minimize tissue trauma, preventing dilator deformation during the procedure, tip displacement and fat necrosis. However, the panel emphasized that these challenges are primarily technique-dependent rather than device selection issues. Hence, there is a need for trained, skilled operators and standardized techniques to overcome these challenges. Regarding paediatric considerations, ports are preferred over other central venous access devices for paediatric patients. Although port blockage rates are higher due to smaller calibre veins, external catheters face greater patency challenges. Ports eliminate the psychological trauma associated with visible catheters and allow for normal activities, including swimming and sports, thereby improving the quality of life during treatment periods.

## 6. What are the Prerequisites for the Insertion of the Port in Terms of Logistics in India?

Thorough planning is essential before port placement in patients with cancer to ensure safety, reduce complications, and maintain long-term device functionality. Key requirements include timely availability of the port device, access to equipped facilities with imaging (ultrasound, C-arm) and monitoring tools, strict adherence to aseptic protocols, and involvement of clinicians or vascular access teams trained specifically in central venous access procedures.[10, 27, 53, 54] Ongoing competency must be maintained through continuous education and periodic skills assessments, for example, through supervised practice and credentialing through dedicated educational programs led by medical and nursing associations.[27, 54] Obtaining informed consent from patients or their guardians, tailored to the patient’s understanding, is both a legal and ethical necessity.[27, 54, 55] Comprehensive documentation, including operator details, specifications, insertion techniques, imaging use, confirmation of catheter tip placement, and procedural or medication changes is necessary. [27, 54] Regular internal audits to monitor complication rates, adherence to protocols, and overall policy compliance are necessary to ensure continuous improvement in care.[27, 54, 56].

A quality improvement project at an Indian tertiary hospital implemented a “care bundle” comprising structured assessments, audiovisual training, and nursing education. During this project, resident doctors observed 30 port-a-cath handling procedures performed by nursing staff across seven patients with solid or haematological malignancies in day care or oncology wards. Following the intervention, improvements were observed in the rate of obtaining verbal consent, pre-procedure preparation of drugs and equipment, adherence to aseptic technique, cross-checking of drug expiry dates, maintenance of port, increased vigilance for signs of extravasation and complete elimination of port-related infections.[57].

Current guidelines do not support routine antimicrobial prophylaxis at the time of central venous port insertion due to risks of allergic reactions and emergence of resistant organisms.[6, 53, 55, 58].

### Indian Expert Panel Consensus:

The prerequisites for the insertion of a port in terms of logistics include (a) availability of the device and access to proper facilities; institutions should be strongly encouraged to have a policy for VAD, including essential equipment and supplies for asepsis, imaging, and monitoring devices, and effective management of biomedical waste; and (b) availability of clinicians or specialized vascular access teams with specific training in venous access procedures. The best practice is to follow image-guided placement (ultrasound guidance for access and fluoroscopic/X-ray guidance to confirm tip position).

### Experts’ Opinion:

Professional societies should establish credentialing standards for the healthcare providers involved in port placement and management. Comprehensive training of the medical personnel and nursing staff is essential. Port access should be restricted to trained staff to ensure patient safety and good treatment outcomes. Regular case auditing should be implemented by hospitals /institutions to monitor outcomes, identify areas for improvement, and ensure adherence to established protocols. Sterile protocols should be implemented when accessing ports because losing ports to infections can be particularly demoralizing for patients. Healthcare systems must ensure easy and quick accessibility to all port sizes, while maintaining cost-effective device options. Patient consent and preprocedural preparation are fundamental requirements. Consent processes should be tailored to the different literacy levels and cultural backgrounds across India. Beyond procedural risks, discussions must also include long-term maintenance, lifestyle changes, and cost implications. Effective interdepartmental coordination and workflow management are required for seamless patient care throughout port placement and maintenance processes. Healthcare teams must have ready access to patients’ medical histories and records to make informed decisions regarding port selection and management. Patient education is crucial for addressing fears about operating theatre procedures and managing complications, such as infection and thrombosis. This involves training patient caregivers, who act as a second set of eyes, to ensure that nurses adhere to sterile protocols when accessing ports. Educational support should encompass patient information leaflets in device packaging, instructional videos, and comprehensive patient-caregiver education programs.

## 7. What are the Prerequisites for the Insertion of other CVADs in Terms of Logistics in India?

Logistical prerequisites for external catheters encompass the availability of devices, trained personnel for insertion and maintenance, sterile environments, suitable storage conditions, necessary equipment such as ultrasound guidance systems, stringent infection control protocols, access to patient medical records, patient consent, and institutional policies and protocols for device care and maintenance.[10, 56, 59] Usually, institutional protocols are developed and validated through close collaboration between medical/surgical oncology and oncology nursing departments. Where protocols are lacking, the barriers most often cited include delays in departmental or hospital-level validation, limited nursing training for PICC insertion, insufficient dissemination of existing guidance, and absence of society-endorsed guidelines.[14].

### Indian Expert Panel Consensus:

The logistical prerequisites for inserting an external catheter include device availability, provider and staff competencies with specific training in VAD management, access to adequate facilities and appropriate equipment, and informed patient consent.

### Experts’ Opinion:

External catheters require intensive ongoing maintenance with frequent dressing changes, line handling, and monitoring. This increases the need for adequately trained nursing staff and a stable staffing ratio. High staff attrition in some facilities poses a significant challenge to sustaining high level of care. To address this, structured training programs with regular certification updates are essential, particularly for the nursing personnel responsible for routine catheter management. Additionally, the higher risk of infection associated with external catheters requires more stringent adherence to aseptic techniques and vigilant monitoring protocols. Such care is also challenging for patients who live at great distances from a medical facility. Institutions should establish clear minimum competency standards and implement standardized checklists and protocols to ensure safe and effective CVAD care.

## 8. Which Factors Determine the Removal of a Port after the Completion of Definitive Therapy?

The decision to remove a port after definitive therapy depends on balancing device complication risks with potential future treatment needs, particularly for cancer patients who may experience recurrence.[9, 10] Some studies recommend removing the port after treatment to avoid complications like device dislodgement and venous thrombosis. [60–62] Port dislodgement rates are 0.3% to 1.5% in adults and 1.4% to 3.6% in children[61] caused by poor port connection, catheter damage, improper positioning, chemical damage from chemotherapy, and mechanical factors like pinch-off syndrome.[61] Port catheter fracture is a rare but serious complication that necessitates port removal. The reported incidence of port catheter fracture varies across studies, with most literature citing rates between 0.1% to 1.8%.[63, 64].

Port retention may be justified in patients who may require further access for follow-up or treatment of recurrence. In one case series of 330 paediatric patients, 144 had ports removed after therapy. Three required re-implantation following leukaemia relapse, occurring after 19–27 months without chemotherapy. Reestablishing central venous access can be challenging in patients with previous central line history.[61] Currently, there is no conclusion about how long a port can be safely retained following the completion of definitive therapy. One study showed 65.7% of patients retained their TIVAD, averaging 973.1 days follow-up (SD = 678; approximately 2.67 years).[65] Studies indicate intravascular adhesion can occur with > 20 months dwell time, particularly in younger patients, those with acute leukaemia and polyurethane catheter use.[60, 66] It has been reported that long-term placement causes difficulty in CVC removal at an incidence rate of 0.3%-2.0%.[53] Because of these factors, the decision regarding port removal should be individualized, considering the patient’s risk of cancer recurrence, the possible complications associated with leaving the port in place, and the feasibility of re-inserting a new port if needed.

### Indian Expert Panel Consensus:

Ports should be removed after the completion of definitive therapy in all patients with a low risk of early relapse and favourable prognosis. However, patients with a high risk of early relapse and an unfavourable prognosis should retain the port with a regular recommended flushing protocol.

### Experts’ Opinion:

The panel agrees that there is significant variation in current clinical practices regarding port management. While some patients retain ports indefinitely as a precaution, others remove them immediately after completing treatment due to fear of complications or desire for normalcy. Infection is the most common cause of unplanned port removal in adult cancer patients, followed by mechanical and thrombotic complications. Therefore, in patients with a favourable prognosis who are unlikely to need more treatment, removing the port can help prevent future device-related complications. However, patients at a higher risk of relapse or needing palliative care should retain their ports. Before deciding on port removal, clinicians should consider the patient’s preference, the risk of future complications, the patient’s ability to attend maintenance visits, available alternatives for vascular access, and any challenges with port reinsertion later if needed.

## 9. How Frequently Should a Central Line be Flushed if not in Use?

CVAD maintenance practices include routine flushing to preserve catheter patency and minimize complications such as thrombotic occlusion and fibrin sheath formation.[4, 67] For inactive ports, flushing intervals may range from 4 to 12 weeks, based on manufacturer recommendations and institutional protocols.[4, 6, 10] One study showed that extending the interval between flushes might be both feasible and safe.. In this cohort, patients with no blood return during port access had a mean interval of 79 days between accesses, versus 63 days among those without difficulty. The difference was not statistically significant (p > 0.05).[68] The authors concluded that monthly port maintenance burdens patients and extending maintenance intervals is safe and beneficial to patients, physicians and healthcare systems.[68] For external catheters, such as tunnelled and PICC lines, weekly flushing remains an accepted standard.[6, 10, 67].

Current evidence does not support the use of prophylactic heparin or other anticoagulants for routine catheter maintenance.[6, 10] Normal saline is considered sufficient for flushing, provided that proper technique including appropriate volume (typically 10–20 mL), pulsatile (“push-pause”) method, and attention to catheter integrity is maintained.[4, 67].

### Indian Expert Panel Consensus:

If not in regular use, ports should be flushed with normal saline every 3 months or according to the instructions for use; external catheters should be flushed weekly. Anticoagulants are not recommended unless they are indicated for other clinical conditions.

## 10. How to Enhance Port Adoption in India?

To enhance port adoption in India, it is essential to address both provider- and system-level barriers, while leveraging the clinical and economic benefits observed with implantable ports. Many oncologists prefer PIVCs and other CVADs due to ease of insertion and perceived cost benefits. Studies show implantable ports offer superior safety profiles, lower complications, and improved quality of life, making them more cost-effective for long-term cancer management.[19, 69] Studies from India and other LMICs confirm that with standard protocols, ports demonstrate safety, efficacy, and longevity, with low rates of infection and other adverse events. [38, 70–72] Barriers in the Indian setting include provider reluctance due to limited training, fear of complications, and lack of equipment like ultrasound and C-arm fluoroscopy.[38] Economic constraints, particularly the high upfront cost of devices and procedures, further limit access, especially for socioeconomically disadvantaged patients.

To overcome these challenges, efforts must focus on increasing awareness and implementing training programs for surgeons and staff, integrating port placement into medical curricula, and implementing standardized protocols.[71] Many hospitals now offer port insertion training programs. Proper staff training and supervision are essential, as poor handling can lead to infections.[38] Hospitals should seek cost reduction methods like combining port placement with primary surgery or performing the procedure under local anaesthesia procedure utilizing day care surgery settings. Policy changes are needed to cover port costs for patients who cannot afford them, with support from patient groups or charities. Education about the benefits and handling of ports is important for both surgeons and patients/caregivers.[38] Awareness must be created that ports are more cost-effective long-term considering utility and reduced complications.

### Indian Expert Panel Consensus:

The panel unanimously agrees that implantable ports are more cost-effective in the long term, despite the initial cost, and result in better overall outcomes for the patient compared to other CVADs. There is a need to increase awareness among healthcare providers and patients with better training and education, as well as the creation of appropriate referral pathways.

### Experts’ Opinion:

The expert panel emphasized that healthcare institutions should develop financial models demonstrating long-term cost-effectiveness despite higher upfront costs. Reliable supply chain management is essential to ensure consistent device availability and competitive pricing. Comprehensive training programs for surgeons, interventional radiologists, and nursing staff across tiers 2 and 3 cities are important. The development of standardized curricula and certification processes is important for ensuring consistent quality standards. Patient education requires addressing misconceptions regarding port placement while emphasizing long-term benefits. Institutions and hospitals play a major role in developing referral pathways between oncologists, surgeons, interventional radiologists and vascular access teams for port insertion and in establishing quality metrics to monitor outcomes and complication rates. Standardized protocols for patient selection, insertion techniques, and post-procedural care should be implemented across hospitals, with institution-specific clinical pathways for port selection, maintenance, and removal to reduce complications and minimize medico-legal risks.

## Data Availability

Data sharing is not applicable to this article as no new data were created or analyzed in this study.
